# Starch-flavonoids interactions from *Cyperus esculentus L.* (tigernut) oil cake: insight on structural, physicochemical properties and digestibility

**DOI:** 10.1016/j.fochx.2025.102999

**Published:** 2025-09-05

**Authors:** Yerkengul Yertai, Zixiang Shao, Yutian Gao, Yidong Wei, Wenting Li, Liqiong Xie, Torkun Mamet

**Affiliations:** a Department of Food Science and Engineering, College of Smart Agriculture, Xinjiang University, Urumqi 830046, China; b College of Life Science & Technology, Xinjiang University, Urumqi 830046, China

**Keywords:** Tigernut, Starch, Flavonoids, Physicochemical properties, *In vitro* digestion

## Abstract

This paper investigated the interaction characteristics between flavonoids and homologous starch in tigernut (*Cyperus esculentus* L.) oil cake. Infrared spectroscopy and X-ray diffraction analyses exhibited that the tigernut flavonoids can bind starch through hydrogen and hydrophobic interactions. SEM and CLSM images revealed a less dense structure in starch-flavonoids complexes, which further confirmed their strong interaction. This interaction increased starch crystallinity, thermal stability and antioxidant properties*,* but reduced the viscoelasticity of the starch (*P* < 0.05). Moreover, the presence of flavonoids significantly strengthened the digestion-resistant characteristic of starch by reducing the content of rapidly digestible starch (RDS) by 30.73 % while concurrently increasing the content of resistant starch (RS) by 21.32 % (*P* < 0.05). The tigernut flavonoids exhibited a slightly greater inhibition of α-glucosidase activity than α-amylase. This research established the theoretical foundations for the development of tigernut flavonoids and starch-based healthy foods and their industrial applications.

## Highlights

• The starch-flavonoids in the tigernut oil cak were bound through non-covalent interactions.
• Flavonoids increased starch crystallinity, thermal stability and antioxidant.
• Flavonoids significantly delayed the digestion of starch.
• Tigernut flavonoid inhibited α-glucosidase activity better than α-amylase.

## Graphical abstract

**Figure:**
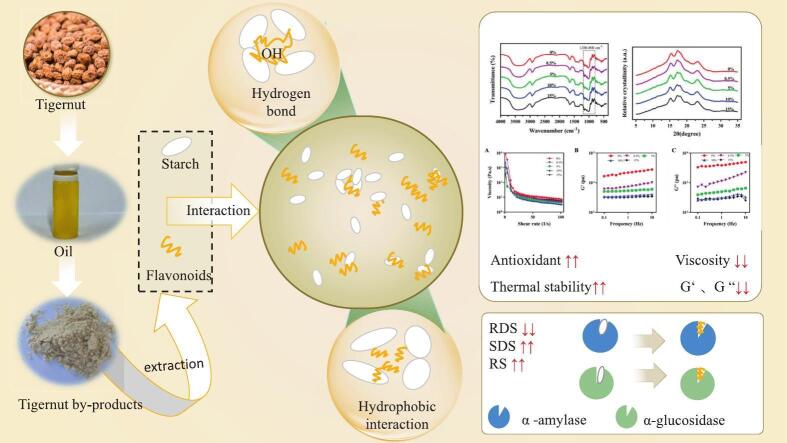

## 1 Introduction

Tigernut (*Cyperus esculentus* L.) is a perennial tuber crop with a sweet nut. It is commonly grown in North Africa, Southern Europe and West Africa due to its ability to grow in acidic soil and arid, sandy terrain (Li et al., 2017). Since the introduction of tigernut to China, they have been widely appreciated across the regions of Xinjiang to improve marginal land use. Tigernut is considered a superior edible oil crop due to its oil rich content of unsaturated fatty acids. Its oleic acid/linoleic acid ratio is similar to that of olive oil, making it a typical healthy oil system of the oleic-linoleic acid type, which can be used as a healthy and nutritious edible oil and contributes to alleviates the shortages of regional grain, oil and energy resources faced by countries around the world (Liu et al., 2019). Meanwhile, tigernut oil cake are abundant in carbohydrates, proteins, cellulose, minerals, and bioactive compounds, and usually used for the production of starch or livestock feeds, but this process may lead to a large loss of bioactive compounds like flavonoids.

Interestingly, previous studies have been shown that tigernut exhibit significant antioxidant and free radical scavenging properties, which may be attributed to secondary metabolites such as flavonoids and phenols components (Edo et al., 2023). Tigernut contain a variety of flavonoids, such as luteolin, quercetin, genistein and kaempferol (Roselló-Soto et al., 2018). It has been discovered that flavonoids possess potent antioxidant activity, antibacterial properties, and digestive enzyme inhibitory activities such as lipase, *α*-glucosidase and *α*-amylase. These effects can assist in reducing insulin resistance and managing diabetes (Llorens et al., 2024). Flavonoids also interact with starch, enhancing nutrition and health benefits, particularly for the development of dietary supplements and functional foods for diabetes patients (Dhital et al., 2019).

Meanwhile, The interaction between starch and polyphenols or flavonoids to control starch digestion and exert anti-diabetic activities has become a hot topic. For example, Li et al. (2024) reported that lingonberry polyphenols interact with corn starch through hydrogen bonding, disrupting the short-range ordered structure of starch, decreasing the thermal stability of starch, and reducing the digestibility of corn starch by generate a higher amount of resistant starch and inhibite the starch enzymes. Huang et al. (2022) discovered that dandelion flavonoids can delay the retrogradation and digestion of potato starch through non-covalent binding. Zhang et al. (2023) showed that the hydrogen bond interaction between chestnut starch and flavonoids delayed the regeneration of starch by inhibiting the formation of long-range ordered crystalline and aggregate structures. The evidence suggests that flavonoids utilize non-covalent bonds as a driving force to enter the internal cavity of starch and form stable complexes. This process manages starch digestion by improving the physicochemical properties of starch, thereby increasing the ratio of resistant starch, or by inhibiting starch enzymes (*e.g.*, α-amylase and α-glucosidase) in the digestive tract (Hu et al., 2023). In addition, recent studies have focused on the interaction between exogenous epigallocatechin gallate (EGCG) and tigernut starch. These studies reveal that their interaction through hydrogen bonding reduces starch crystallinity, thermal stability, viscosity, and gel elasticity, whereas enhancing starch solubility, freeze-thaw stability, and resistant starch content (Li et al., 2025). However, the effects of flavonoids on starch digestion in tigernut oil cake, as well as their interactions are inconclusive.

Conventionally, flavonoids and starch from tigernut oil cake are considered high-value components that hold great potential for significantly enhancing the recycling efficiency and economic value of this agricultural by-product. Therefore, this study aims to reveal the interaction mechanism between flavonoids and starch from tigernut oil cake, and to analyze their effects on the structure, physicochemical and digestibility properties of tigernut starch. To this end, the changes in the ordered structure, crystal structure and microstructure of starch after the addition of flavonoids were analyzed by Fourier transform infrared spectroscopy (FT-IR), X-ray diffraction (XRD), scanning electron microscopy (SEM) and confocal laser scanning microscopy (CLSM). The impact of the interaction on the antioxidant, thermal, rheological, and digestive properties was then examined. The inhibitory effects of flavonoids on starch digestive enzymes including α-amylase and α-glucosidase, were also studied. This will provide a useful reference for improving the processing of tiger nut functional products.

## 2 Materials and methods

### 2.1 Materials

Tigernut was purchased from the 54 th Regiment of the Third Division, Xinjiang Production and Construction Corps (XPCC), China. The tigernut oil cake was produced through a laboratory-scale enzymatic oil extraction process, which utilized a composite enzyme system (cellulase: pectinase: alkaline protease), followed by centrifugation and drying.

Porcine pancreatic α-amylase (50,000 U/g), and α-glucosidase (100,000 U/g) were purchased from Shanghai Yuanye Biotechnology Co., Ltd. (Shanghai, China). Fluorescein isothiocyanate (FITC) was purchased from Solarbio (Solarbio Biotechnology Co., Ltd. Beijing, China). 3,5-Dinitrosalicylic acid (DNS) was purchased from Coolaber. All other chemicals reagents used in this study were of analytical grade and all solutions are prepared using distilled water.

### 2.2 Preparation of samples

The flavonoids from tigernut by-product was prepared as described by Rosello-Soto et al.(2018). The powdered samples (1 g) of the tigernut tuber after oil extraction (aqueous enzymatic extraction) added to 15 mL ethanol (25 %, *v*/v) and it was stirred on a plate with a magnetic stirrer at 50 °C for 3 h. Cover the sample with aluminum foil. The obtained sample was filtered, concentrated, freeze dried. The total flavonoid content of the tigernut oil cake was 15.09 mg /100 g.

The powdered samples (100 g) of tigernut oil cake after flavonoids extraction were extracted in distilled water at ratio of 1:5 (g/mL), centrifuged at 6690 rpm for 20 min until white precipitate was obtained, and then dried at 40 °C for 24 h. The purity of resulting starch fractions was 86.60 %, and the amylose content was 19.65 %.

### 2.3 Preparation of the starch-flavonoids complex

The preparation of starch-flavonoids was according to the method of Hu et al. (2023). Mix the tigernut flavonoids (mass fraction: 0 %, 0.5 %,5 %,10 %,15 %, based on dry weight of starch) with a uniform starch slurry (12 %, *w*/w). The mixture was placed on a magnetic stirrer at 50 °C and stirred at 260 rpm for 2 h. And then centrifuged at 2000 rpm for 15 min. The precipitate was freeze-dried, crushed, sieved, and sealed spare.

### 2.4 Fourier transform infrared (FT-IR) spectroscopy

The infrared spectra of the complexes were scanned from 500 to 4000 cm^−1^ by using FTIR (VERTEX 70 RAMI, Bruker, Germany). The ratios of R 1047/1022 cm^−1^ and R 995/1022 cm^−1^ were calculated by using Omnic 8.0 software.

### 2.5 X-ray diffraction (XRD) analysis

The crystalline characteristics of the complexes were analyzed by using an X-ray diffractometer (D8 ADVANCE, Bruke, Germany). The sample was scanned at a speed of 4°/min, scanned from 10°-40° (2θ), accompanied by a scanning step of 0.02°. Complexes relative crystallinity (RC %) was calculated using the Jade 6.0 software.

### 2.6 Scanning electron microscopy (SEM)

The microstructure of the starch-flavonoid complex was characterized using a scanning electron microscope (SU8010, Hitachi, Japan). Approximately 5 mg of sample was uniformly adhered to conductive carbon tape and sputter-coated with a thin gold layer prior to imaging at 5 kV accelerating voltage under 10,000× magnification.

### 2.7 Confocal laser scanning microscopy (CLSM)

The microstructure of the complexes was observed using a confocal laser scanning microscope (OLS5100-SAF, Nikon, Germany) following the previously described procedure (Zhou et al., 2015). Starch pastes (4 %, *w*/w) prepared using the Rapid Visco Analyzer apparatus. After 48 h storage, the samples were stained with 2 mg/mL fluorescein isothiocyanate (FITC) for 24 h to observe the position of starch. Placed the stained sample on a glass slide, covered with a cover slip, and observed under 488 nm excitation.

### 2.8 Rheological properties

The rheological properties of starch-flavonoids complex pastes were measured in a non-destructive manner using a rotational rheometer (HAAKE MARS60 + iS50, China). A plate with a 35 mm diameter and a 1.0 mm gap was chosen, with the test temperature set at 50 °C and a uniform sample equilibration time of 30 s for all measurements. 1.0 g of starch-flavonoids complex was dispersed in distilled water to form a uniform suspension (10 % *w*/*v*). Then, it was heated at 85 °C for 5 min to obtain the starch paste (Punia et al., 2019). The sample immediately loaded on the ram of the rheometer. Afterthat, a cover plate was employed to cover the samples tested to minimize heat loss and evaporation during measurements. For steady state, the shear rate was increased from 0.1 s^−1^ to 100 s^−1^ at a strain of 1 % (in the linear viscoelastic region). The apparent viscosity as a function of shear rate was obtained (Wang et al., 2018).

Subsequently, the dynamic oscillatory frequency scanning test was conducted at frequencies in ranging from 0.1 Hz to 10 Hz, and the storage modulus (G') and loss modulus (G") of starch-flavonoids were recorded (Hu et al., 2023).

### 2.9 Differential scanning calorimeter (DSC) analysis

The thermal characteristics of the complexes were analyzed by using a differential scanning calorimeter (TA Instruments DSC2500). Accurately weigh 3.0 mg (dry basis) of the sample and 6.0 mg of deionized water in an aluminum pot, sealed it, and balanced it at 25 °C for 24 h, then heated it from 25 °C to 120 °C at a speed of 10 °C/min.

### 2.10 Evaluation of antioxidant capacity

To evaluate the antioxidant activity of the starch-flavonoids complex (0 %–15 %), the DPPH radical scavenging assay and reducing capacity assay were carried out based on the method of Ma et al. (2023), with minor modifications.

The DPPH radical scavenging capacity test. Starch-flavonoids samples (4 mg/mL, 0 %–15 %) were mixed with 4 mL of DPPH solution (2 × 10^−4^ mol/L). For controls, samples were replaced with an equal volume of ethanol. Next, the mixture was centrifuged for 10 min at 7000 rpm. The absorbance of the supernatant was recorded at 517 nm after the reaction for 30 min under conditions that avoided light. Calculate the percentage of DPPH scavenging activity according to the following formula:

(1) $$\mathrm{DPPH radicalscavenging}\left(\%\right)=\left[1-\frac{A_{\mathrm{control}}-\left(A_{\mathrm{sample}}-A_{\mathrm{black}}\right)}{A_{\mathrm{control}}}\right]$$

where A_control_ is the absorbance of the control (ethanol instead of sample), A_sample_ is the absorbance of the sample, and A_black_ is the absorbance of the sample only (ethanol instead of DPPH).

The reducing power test. Starch-flavonoids samples (5 mg/mL, 0 %–15 %) were mixed with 0.2 M sodium phosphate buffer (1.25 mL, pH 6.6) and 1 mL of 1 % (*w*/*v*) potassium ferricyanide. The mixture was incubated at 50 °C for 20 min with shaking, then centrifuged at 6000 rpm for 5 min after adding 1.25 mL of 10 % (w/v) trichloroacetic acid. The supernatant (2.5 mL) was combined with distilled water (2.5 mL) and 0.1 % (w/v) ferric chloride (0.5 mL), and absorbance was measured at 700 nm.

### 2.11 *In vitro* starch digestion

The digestibility of starch-flavonoids was measured according to Ren's et al.(2019) method with minor modifications. Firstly, the 0.05 g of starch-flavonoids complexes were weighted and dissolved in 5 mL of sodium acetate buffer (0.2 M, pH 5.2). Next, 5 mL of the digestive enzyme mixture (5 U/mL α-amylase and 0.5 U/mL α-glucosidase) was added. Thereafter, the mixture was oscillated continuously at 150 rpm in a 37 °C water bath. At predetermined time intervals (20, 30, 60, 90, 120, and 180 min), 1 mL aliquots of the hydrolysate were withdrawn and immediately mixed with anhydrous ethanol (5 mL) to inactivate the enzymes. The mixtures was centrifuged at 3000 rpm for 10 min at 4 °C, and the supernatants (1 mL) was transferred to clean test tubes. Each supernatant was mixed with 1 mL of DNS reagent, boiled for 5 min, cooled to room temperature, and diluted to a final volume of 50 mL with deionized water. The absorbance was measured at 520 nm. The contents of RDS (rapidly digestible starch), SDS (slowly digestible starch), and RS (resistant starch) were calculated by the following equation:

(2) RDS=0.9×G20×100%

(3) $$\mathrm{SDS}=0.9\times \left(G120-G20\right)\times 100\%$$

(4) RS=100−RDS−SDS

where G20 and G120 are the released glucose mass after 20 min and 120 min hydrolysis (mg/mL), 0.9 is the conversion factor.

### 2.12 α-Amylase and α-glucosidase inhibition

Enzyme (α-amylase and α-glucosidase) inhibition assay were carried out based on the method of Yu with minor modifications (Yu et al., 2024). Tigernut flavonoids complex was used to measure the inhibitory activity on the enzyme. Percent inhibition was calculated by:

α-Amylase activity test. The tigernut starch-flavonoids complex prepared in Section 2.3 was dissolved in phosphate buffer (0.2 M, pH 6.8) to a concentration of 0.5 %. Then, heated at 80 °C for 30 min to ensure complete dissolution and stabilization. The different starch-flavonoids solution (300 μL) was mixed with α-amylase (3.75 U/mL, 100 μL) into a glass tube with plugs, and vibrate the reaction at a speed of 150 rpm in 37 °C water bath for 10 min. Next, the reaction was terminated by add 1 mL of 3,5-dinitrosalicylic acid (DNS) reagent, followed by boiling for 5 min. After rapid cooled to room temperature, the volume was adjusted to 25 mL with deionized water. Finally, measured the absorbance at 540 nm. The inhibition rate (%) was calculated by the following formula:

(5) $$\text{Inhibition rate}\left(\%\right)=\left[1-\frac{\left(A_{\mathrm{sample}}-A_{\mathrm{control}}\right)}{\left(A_{\mathrm{blank}}-A_{\mathrm{blank}-\mathrm{control}}\right)}\right]$$

where *A*_*sample*_ and *A*_*control*_ refer to the absorbance of the flavonoids and non- flavonoids treated samples respectively; *A*_*blank*_ and *A*_*blank-control*_ are the sample group and control group.

α-Glucosidase activity test. The tigernut starch-flavonoids complex prepared in step 2.3 was dissolved in phosphate buffer solution (0.2 M, pH 6.8) to reach a concentration of 0.5 %. Then, the starch-flavonoids solution was mixed with 50 μL of α-glucosidase (0.5 U/mL) in a microcentrifuge tube and incubated at 37 °C for 10 min in a shaking water bath. After that, 100 μL of pNPG (3 mmol/L) solution was added and the reaction was carried out at 37 °C for 30 min. Then, 750 μL of Na_2_CO_3_ (0.1 mol/L) solution was immediately added to terminate the reaction. Finally, measured the absorbance at 450. The α-glucosidase inhibition rate was calculated using formula (4).

### 2.13 Statistical analysis

All the experiments were determined in triplicates. Experimental data were analyzed using an analysis of variance (ANOVA) and Duncan's test (*P* < 0.05) by SPSS software (version 16.0, IBM). GraphPad Prism software (9.5.0, USA) was used to perform construct graphs.

## 3 Results and discussion

### 3.1 Interaction mechanism of tigernut starch-flavonoids

**Fig. 1:**
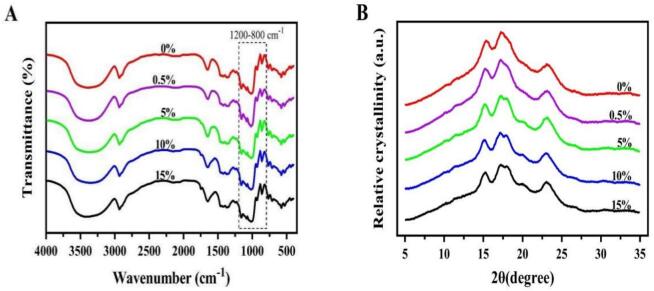
FT-IR (A) and X-ray diffraction (B) analysis of tigernut starch-flavonoids complexes. The concentration gradients (0 0 %–15 %) denote the mass ratios of flavonoid addition to starch-flavonoid complexes.

Fig. 1A illustrates the FT-IR spectra of tigernut oil cake starch-flavonoids complexes, to detect the functional groups and short-range organized structures. All the samples exhibited a broad absorption band at 3700–3000 cm^−1^, which is attributed to the O—H stretching vibration of the starch hydroxyl groups (Wang et al., 2018). The absorption peaks of this band became broader with addition of flavonoids concentration. Furthermore, the introduction of flavonoids redshifted the absorbance peaks from 3417 cm^−1^ to 3375 cm^−1^ for tigernut starch, which indicates stronger hydrogen bonds between starch and flavonoids (Huang et al., 2022). In addition, the intensities of absorption peaks at 2950 cm^−1^ (CH_2_ asymmetric stretching) and 1640 cm^−1^ (flavonoid C=O stretching) decreased upon flavonoid incorporation (Hu et al., 2023), demonstrated that there was an interaction between the hydroxyl groups of flavonoid and the C—H group in the complexes (Raza et al., 2023). These findings align with previous reports on non-covalent interactions driving starch-flavonoids (Hu et al., 2023; Huang et al., 2022). Notably, no new characteristic peaks emerged in starch-flavonoids complexes compare to the native starch, confirming the absence of covalent bond formation (Li et al., 2024). Overall, these results indicate that the driving force behind the interactions between tigernut oil cake starch and flavonoids is primarily non-covalent in nature.

The spectra were deconvoluted from 800 to 1200 cm^−1^ to further study the effect of flavonionds on the short-range ordered structure of tigernut oil cake starch. The absorption peaks at 1047 cm^−1^ and 1022 cm^−1^ were associated with the crystalline and amorphous regions of starch, and the absorption peak at 995 cm^−1^ was related to the intermolecular hydrogen bonding within the helical structure of hydrated carbohydrates (Monroy et al., 2018). Generally, the ratio of peak at 1047 cm^−1^ and 1022 cm^−1^ (R_1047/1022_) can reflect the degree of order of starch structure and the interactions between starch molecular chains (Wang, Wu et al. 2022). The ratio of 995 cm^−1^ to 1022 cm^−1^ (R_995/1022_) is related to the degree of the double-helix structure of starch and the hydrophobic interactions between starch and flavonoids (Zhou et al., 2021). As shown in Table 1, compared with the control group (0 %), the R_995/1022_ increased from 1.13 to 1.18 (*P* < 0.05), indicating a potential hydrophobic interaction between starch and flavonoid compounds. This finding is consistent with the study by Hu et al. (2023), which reported that flavonoids can bind to short-chain starch molecules through hydrophobic interactions, inhibiting recrystallization and ordered structure formation while enhancing short-range structural order. Furthermore, the change in R_1047/1022_ was not significant, but the ratio increased to 0.56 when 15 % flavonoids were added. Combined with the results of FT-IR, it can be inferred that there may exist a weak hydrogen bonding interaction between starch and flavonoids. Additionally, these ratios reflect short-term molecular rearrangements within starch granules and correlate with starch crystallinity to a certain extent (Liu et al., 2020), as will be further discussed in subsequent results. Collectively, the FT-IR results indicate that the binding of tigernut oil cake starch-flavonoids complexes was driven by hydrogen bonds and hydrophobic interactions.

**Table 1:** FT-IR and X-ray diffraction analysis of tigernut starch-flavonoids complexes.

	0%	0.5 %	5 %	10 %	15 %
R1047/1022 cm^−1^	0.55 ± 0.01^ab^	0.54 ± 0.00^b^	0.55 ± 0.00^a^	0.55 ± 0.00^a^	0.56 ± 0.02^a^
R995/1022 cm^−1^	1.13 ± 0.02^c^	1.05 ± 0.00^d^	1.20 ± 0.00^a^	1.17 ± 0.00^b^	1.18 ± 0.00^ab^
Relative crystallinity (%)	9.45 ± 00^d^	11.62 ± 00^c^	13.18 ± 00^b^	13.41 ± 00^b^	15.68 ± 00^a^

The concentration gradients (0%–15%) denote the mass ratios of flavonoid addition to starch-flavonoid complexes. Different superscript lowercase letters in the same line mean significantly different (*P* < 0.05).

The crystal structure of starch-flavonoids complexes was analyzed *via* X-ray diffraction analyses. As shown in Fig. 1B, the characteristic peaks of tigernut oil cake starch appeared at 13°, 17°, and 23°, showing typical A-type starch (Liu et al., 2021). With the increasing addition of flavonoids, the characteristic peaks of A-type starch in the complex disappeared, while the characteristic peaks of the V-type complex appeared around 20° (Gutiérrez et al., 2022). Furthermore, as shown in Table 1, with the increase in flavonoids concentration, the crystallinity of starch significantly improved. The incorporation of 15 % flavonoids increased the crystallinity of starch by approximately 6.23 % compared to that of natural starch (*P* < 0.05), indicating that flavonoids can promote the formation of crystalline structures in starch. This is consistent with the findings with Wang et al. (2021), but contradicts the result found by Zhu et al. (2012) that starch and rutin cannot form a V-shaped complex. This may be related to differences in starch varieties, flavonoid types, structures and concentrations. The interaction between starch and flavonoids compounds occurs through the formation of V-type inclusion or non-inclusion complexes (Li et al., 2024). It has been also reported that the driving force for the formation of amylose clathrates is the hydrophobic interaction within the helical cavity (Wang et al., 2024). Since the flavonoids in tigernuts are predominantly flavonols (such as quercetin, orientin, *etc.*), their planar and rigid structures make them more conducive to embedding into the helical cavity of starch (Edo et al., 2024). Therefore, it can be inferred that the hydrophobic chain moieties of flavonoid compounds bind to starch, or that flavonoid compounds embed into the helical cavity of amylose through hydrophobic interactions, thereby forming V-type complexes. Meanwhile, it is evident that flavonoid compounds form hydrogen bonds with the hydroxyl groups of starch, which may also lead to an increase in crystallinity (Wang et al., 2024), this is align with the FT-IR results. These results suggest that tigernut flavonoids bind to homologous starch *via* hydrogen bonding and hydrophobic interactions, leading to the formation of stable V-type starch-flavonoid complexes.Thus, the flavonoids and starch derived from oil cake have emerged as potential additives or matrices of high nutritional value in managing metabolic syndrome (Gutiérrez et al., 2022).

### 3.2 Morphological changes in tigernut starch- flavonoids complexes

**Fig. 2:**
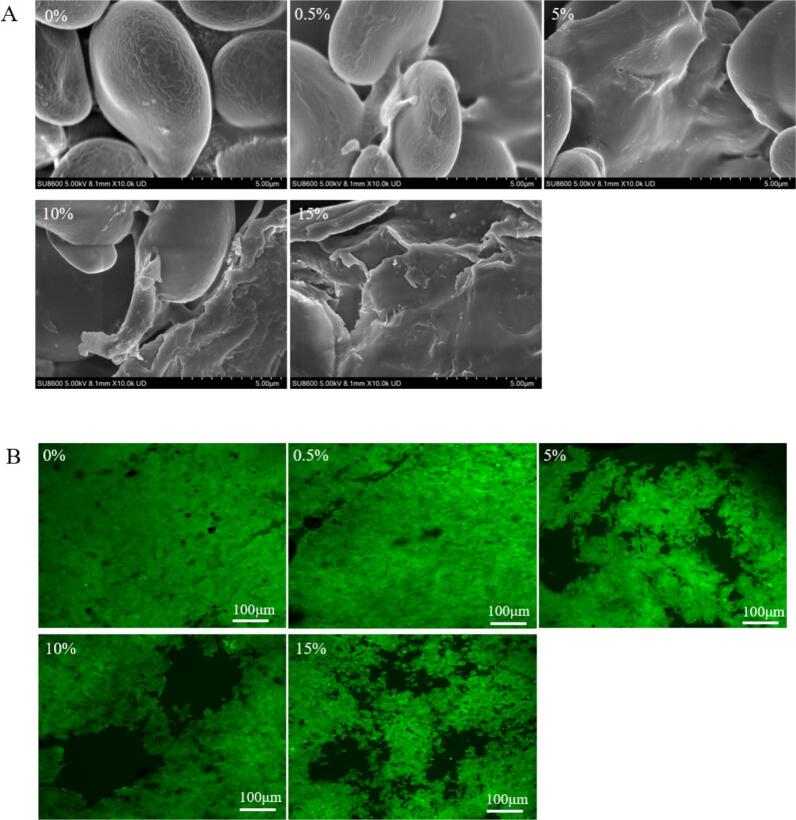
SEM image (A) and CLSM images (B) of the starch-flavonoids complexes. The concentration gradients (0 0 %–15 %) denote the mass ratios of flavonoid addition to starch-flavonoid complexes.

SEM micrograph was used to observed the changes in the surface morphology of tigernut oil cake starch caused by flavonoids. The images showed that tigernut oil cake starch was dominated by elliptical starch granules with uneven sizes and relatively smooth surfaces (Fig. 2A). However, with the concentration of flavonoids increases, the more fine-needle-like substances adhered and accumulated disorderly on starch surface, and the starch became less continues. The losser structure of starch-flavonoids complexes is in line with the previous research results (Huang et al., 2022; Yang et al., 2019). In addition, flavonoids can cross-link and aggregated with starch molecules. Huang et al. (2022) discovered that the hydroxyl structure of flavonoids can combine with starch through hydrogen bonds, resulting in a looser gel structure of starch by preventing the formation of hydrogen bonds between starch molecules. Thus, these studies suggested that the introduce of flavonoids alter the microstructure of natural starch particles, transforming their smooth surfaces into rough, porous, loose or aggregated states, leading to the modification of the starch network and thereby affecting their structural properties, gel properties, digestibility and texture stability. This phenomenon will be further discussed in CLSM.

As depicted in CLSM images (Fig. 2B), for native starch, forming a continuous and glutinous network. It was confirmed that amylose is leached from the starch particles, and then the starch is swollen and even decomposed, producing cloud-like surroundings with a typical starch-gel fiber network cascading (Wang et al., 2018). The tigernut oil cake starch-flavonoids complexes exhibited a different arrangement, compared with native starch. As the flavonoids concentration increases, the starch components were less likely to overflow from the starch particles. Some swollen starch granules tend to aggregate, and apparent cloud-like surrounding, which is in consistent with Wang et al. (2018), where lotus leaf flavonoids distributed around starch granules and creating cloud-like surrounding. Additionally, for fluorescein isothiocyanate (FITC), affinity is largely equivalent to hydrophobicity, FITC accumulates in hydrophobic regions (Zhou et al., 2015). It was hypothesized that the flavonoids would interact with the leached amylose, retaining more water within the complex, reducing the amount adsorbed by the starch particles, and forming a denser gel with a more compact starch structure (Lyu et al., 2022). Therefore, the hydrophobic and hydrogen bond interactions in starch-flavonoids complexes lead to significant changes in the microstructure of starch.

### 3.3 Rheological properties

**Fig. 3:**
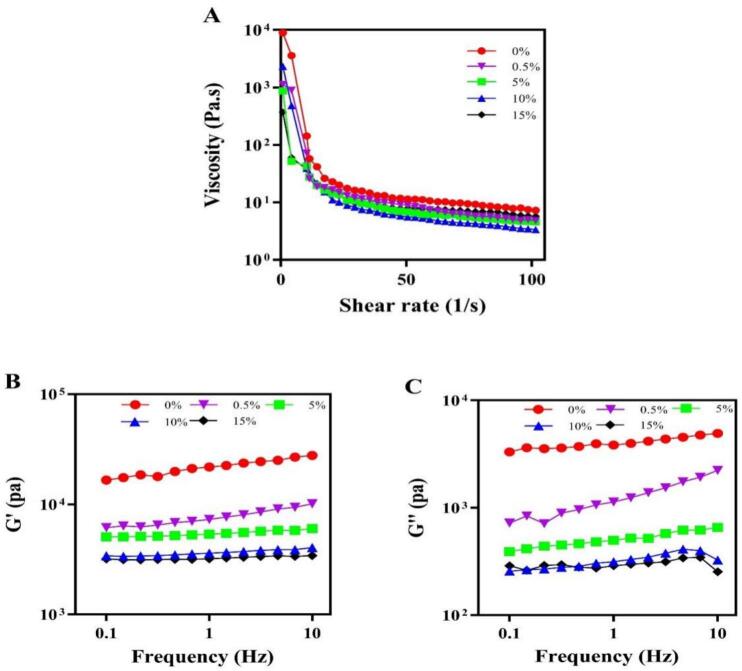
The rheological properties of tigernut starch-flavonoids complexes, including viscosity (A); G′-storage modulus (B); and G′′-lost modulus (C). The concentration gradients (0 0 %–15 %) denote the mass ratios of flavonoid addition to starch-flavonoid complexes.

The rheological properties of starch serve as a core indicator for evaluating its processing applicability and structural stability. In this study, the impacts of tigernut flavonoids compounds on the rheological behavior of homologous starch were elucidated through static rheology (shear-rate sweep) and dynamic rheology (frequency sweep). Static rheology was employed to investigate the variation pattern of starch viscosity in the tigernut oil cake starch-flavonoids interaction under different shear rates, as depicted in Fig. 3 **A**. The viscosity of all samples decreased sharply with an increase in shear rate and then leveled off, exhibiting typical shear-thinning behavior and pseudoplastic fluid characteristics (Li et al., 2020). However, the introduction of flavonoids resulted in the viscosity of all starch-flavonoid interactions being consistently lower than that of the control starch (0 %), attributing these reductions to the possible formation of starch-flavonoids and the competitive effect of flavonoids with starch granules for water, which reduced the elastic properties and flow resistance of the starch (Huang et al., 2022). Meanwhile, flavonoids coated the surface of starch particles to reduce the degree of swelling (Wu et al., 2021). The change in flow behavior could be attributed to the structures of amylose and the presence of flavonoids. The addition of flavonoids weakened the internal entanglement of the amylose double helix network, leading to changes in the starch gel network and thereby reducing the starch viscosity (Wang et al., 2022). This result revealed that flavonoids exhibited a protective effect against the swelling of starch particles.

The effect of the interaction between starch and flavonoids on the dynamic rheological properties was also studied. The values of the energy storage modulus (G') and the lost modulus (G") were shown in Fig. 3**B** and **C**. Both the moduli of all samples increased progressively, regularly with the increasing of frequency, and there was no intersection between G′ and G′′, demonstrated that the starch paste was relatively stable within the observed range. Furthermore, all the G′ values were higher than the corresponding G′′ values across the measured frequency range, and both moduli exhibit a great frequency dependence, suggested that all samples were typical weak gel pastes, with starch's elastic behavior dominating (Wang et al., 2021). The presence of flavonoids caused a decline in the G′ and G″ compared to the native tigernut starch. This is consistent with previous reports (Chávez-Santoscoy et al., 2016), the introduction of flavonoids into the starch could reduce the elastic properties and flow resistance of starch. However, Hu et al. (2023) found that bamboo leaf flavonoids addition led to the enhanced both modulus. This difference is that the degree of this disruption in rheological properties is influenced by the flavonoids type, concentration and interaction type with starch. Therefore, the addition of tigernut flavonoids can disrupt the continuity of the starch network, decrease the junction region of starch molecules, and the hydrogen bonds formed in the complex interfere with the formation of amylose double helix, resulting in a lower modulus and apparent viscosity (Huang et al., 2022). These results underscore flavonoids' role in modifying starch rheological properties, providing insights into its potential for starch-based products.

### 3.4 Evaluation of thermal properties and antioxidant capacity

**Fig. 4:**
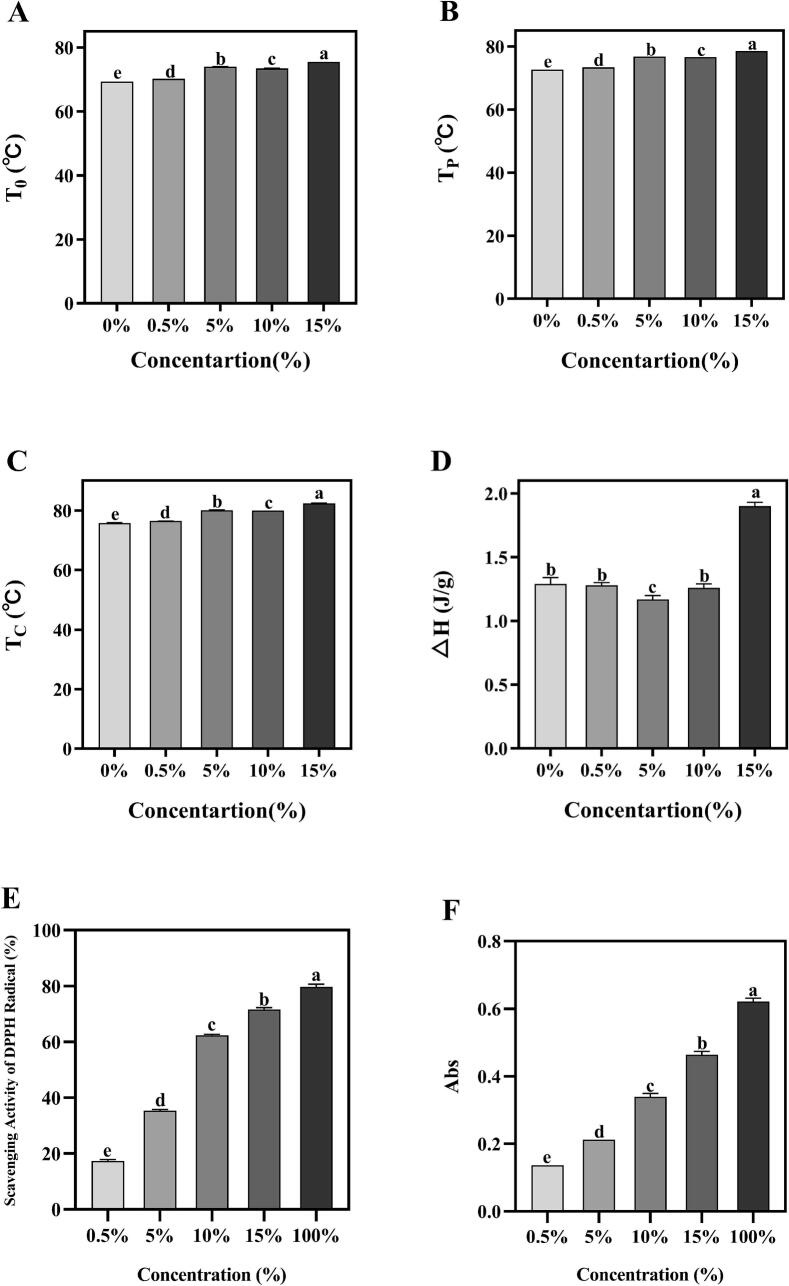
The thermal properties (A: To; B: Tp; C: Tc; D: ΔH) and antioxidant capacity (E: DPPH scavenging capacity; F: the reducing power) of tigernut starch-flavonoids complexes. The concentration gradients (0 0 %–15 %) denote the mass ratios of flavonoid addition to starch-flavonoid complexes. Different lowercase letters indicated significant differences among groups (*P* < 0.05).

The thermal properties of starch in the presence of flavonoids were observed in Fig. 4A-D. Compared with the tigernut oil cake original starch, the gelatinization temperature and enthalpy change of starch significantly increased with increasing flavonoids content. The *T*_*0*_ and *T*_*P*_ values of the 15 % flavonoids group increased by about 6 °C compared with the control, while the ΔH values of starch raised significantly with flavonoids. This result indicated that the double helix chain length of starch-flavonoids complexes was longer (Li et al., 2018), and the hydrophobic interaction between flavonoids and starch limited water penetration into the amorphous regions of starch, making swelling, cracking and adhesion more difficult. This is agree with previous reported, who observed that tea polyphenols significantly enhanced the thermal properties (gelatinization enthalpy and temperature) of wheat starch (Zhang et al., 2015). The change could be attributed to the fact that the hydrophilic OH groups of flavonoids may bind to starch particles by hydrogen bonds and hydrophobic interactions, enhancing the crystallinity and ordered structure of starch and forming stable complexes. Consequently, a higher temperature is required to disrupt the double helix and crystalline structure of the starch. These results further confirmed that the interaction in starch-flavonoids complexes leads to increased resistance to starch gelatinization during heating. The DSC results were consistent with XRD (Fig. 1B). However, the results contradicted previous findings that *Vaccinium bracteatum* Thunb. leaf pigment decreased the gelatinization temperature of rice starch (Xu et al., 2017). This might be caused by differences such as the double helix structure and crystallinity of starch, and the polyhydroxyl structure of flavonoids. These results indicated that the starch-flavonoids complexes protected the ordered structure and thermal properties of tigernut starch. This characteristic makes starch-based foods advantageous in high-temperature processing applications such as baking, squeezing and frying.

Furthermore, to detect the antioxidant capacity of the starch-flavonoids complexes, the DPPH free radical scavenging capacity and the reducing power assay were determined. As shown in Fig. 4E, the DPPH scavenging activity of the starch-flavonoids showed excellent DPPH scavenging activity in a concentration-dependent manner, especially at 15 % flavonoid addition (71.51 %), while native starch (0 % flavonoid addition) showed lower scavenging activity. The results indicated that starch can interact with flavonoids to improve the antioxidant capacity of the starch-flavonoids complexes. Similar results are also observed in Xu et al. (2021) and Ma et al. (2023), who found that the scavenging activity of DPPH is positively correlated with the content of phenolic substances in the CMCS-based conjugate and the well scavenging capacity is mainly relied on the morin moieties linked with the amylose backbones. Moreover, the reducing power assay also showed similar results to DPPH test (Fig. 4F). Compared with the starch, the antioxidant capacity of starch-flavonoids complexes was significantly improved in a concentration-dependent. This indicated that flavonoids concentration may be the main factors to affecting the antioxidant capacity of starch-flavonoids complexes (Bai et al., 2020). The result is consistent with the previous work (Ma et al., 2023), starch can enhanced the hydrogen-donating ability of hydroxycinnamic acids due to its conjugated electron-donating effects, suggested the antioxidant capacity of the complexes was improved by a non-covalent. The results demonstrated that hydrogen bonds exist between starch molecules, and flavonoids were firmly connected to the starch chain. Consequently, the antioxidant capacity of the complexes was enhanced with the addition of flavonoids.

### 3.5 Effect of flavonoid on the digestibility of starch *in vitro*

**Fig. 5:**
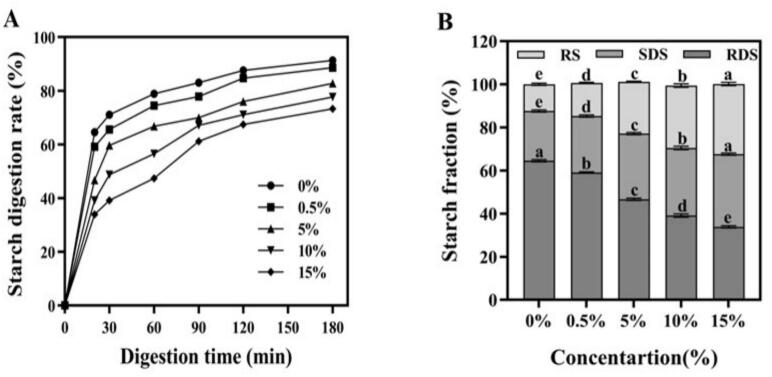
*In vitro* digestibility (A) and composition (B) of starch. The concentration gradients (0 0 %–15 %) denote the mass ratios of flavonoid addition to starch-flavonoid complexes. Different lowercase letters indicated a significant difference among groups (*P* < 0.05).

As shown in Fig. 5A, all samples were gradually digested with the extension of digestion time, and much of starch were enzymatically digestion in the first 90 min, plateauing at 120 min. The interaction of tigernut flavonoids and starch prevent the hydrolysis of starch in a dose-dependent manner. Furthermore, the rapidly digestible starch (RDS), slowly digestible starch (SDS) and resistant starch (RS) contents of starch-flavonoids complexes are displayed in Fig. 5B. Tigernut flavonoids obviously restrained the starch digestibility, and its inhibitory effect increased with the flavonoids concentration. The RDS content in the starch significantly decreased from 64.57 % to 33.87 %, as the RS content increased from 12.40 % to 32.52 % with a 15 % flavonoid substitution. Simultaneously, the incorporation of 15 % flavonoids also resulted in an approximate 10.77 % increase in SDS content compared to the native starch. Generally speaking, foods with lower viscosity, less dense microstructure and softer texture are more conducive to structural decomposition and enzymatic hydrolysis during digestion (Huang et al., 2022). The introduction of flavonoids decreased the viscosity of the starch; however, starch digestion was also hindered. These results further verified the strong interaction between flavonoids and starch. Ren et al. (2019) also reported that the molecular structure of amylopectin provides a strong interaction between polyphenols and starch, resulting in polyphenols can bind with starch through hydrogen bonds and hydrophobic interactions, which would retard starch digestion and increase RS content. Similarly, Hu et al. (2023) have shown that bamboo leaf flavonoids adhere to the surface of pea starch granules and form aggregates, thereby reducing the contact area between digestive enzymes and starch grains. These outcomes indicated that the high-molecular-weight tigernut flavonoids can significantly inhibit the digestibility of starch and enhance the RS content. This agrees with the aforementioned structural changes, where starch and flavonoids form a stable and ordered structure, leading to the aggregation of starch molecules. This, in turn, creates a physical barrier against enzymatic digestion, inhibiting the digestibility of starch.

### 3.6 Inhibition of α-amylase and α-glucosidase activity by flavonoids

**Fig. 6:**
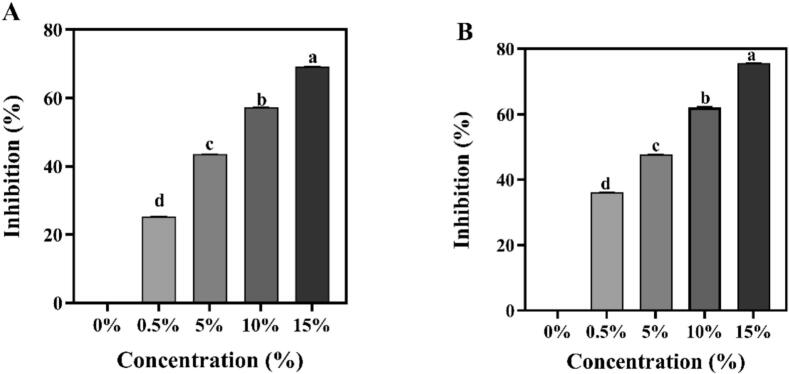
Inhibition of α-amylase (A) and α-glucosidase (B) activity by different concentrations of flavonoids. The concentration gradients (0 0 %–15 %) denote the mass ratios of flavonoid addition to starch-flavonoid complexes. Different lowercase letters indicated a significant difference among groups (*P* < 0.05).

The α-amylase and α-glucosidase control the pathways of hyperglycemia by regulating starch digestion. The effects of tigernut flavonoids complexes on α-amylase and α-glucosidase inhibition are depicted in Fig. 6**A** and **B**. The starch-flavonoids complexes could cause significant α-amylase and α-glucosidase inhibition at low concentration of flavonoid (0.5 %). In addition, there is an increased linearly relationship between the inhibition activity and the concentrations of flavonoids in starch-flavonoids (Yu et al., 2024). The most effective enzyme inhibition rates for α-amylase and α-glucosidase were 83.50 % and 93.48 % at the concentration of 15 % flavonoid, respectively. This study exhibited that flavonoids more significantly inhibited α-glucosidase activity than α-amylase activity. Thus, it is suggested that the flavonoid extract of tigernut was a mild α-amylase inhibitor but a strong α-glucosidase inhibitor (Li et al., 2024). Moreover, many previous studies have reported that different molecular structures of phenolic acids have different inhibitory effects on starch-digesting enzymes. These results demonstrated that there was strong non-covalent interaction between tigernut flavonoids and starch, which suppresses enzyme activity and reduces starch digestibility.

Therefore, the low digestibility of the tigernut starch-flavonoids complexes system may be caused by two mechanisms: (1) flavonoids and starch form a more complex network structure through non-covalent interaction, resulting in higher short-range molecular structure and higher crystallinity. (2) flavonoids with high molecular weight and high complexity adhere to the starch surface, and some of the free flavonoids in the complex limiting the accessibility of amylase, thus inhibiting the digestion of starch (Li et al., 2018; Ren et al., 2019; Wang et al., 2025).

## 4 Conclusions

The interaction between flavonoids from tigernut oil cake and homologous tigernut starch was studied. Flavonoids bind to starch improved the order, crystallinity, thermal stability and antioxidant properties of starch, but decreased the gel viscoelasticity of starch. This changes in these physicochemical properties might be responsible for the flavonoids interact with starch through hydrogen bonds and hydrophobic interactions. In addition, the changes in tigernut starch structure caused by the presence of flavonoids are one of the reasons for the inhibition of starch digestibility. Further, tigernut oil cake starch-flavonoids complexes inhibits α-amylase and α-glucosidase activities. Overall, the interactions between starch and flavonoids in tigernut oil cake have revealed the potential utilization value of tigernut as a thermally processable functional food, providing a theoretical basis for the comprehensive utilization of tigernut. Future research should systematically validate the regulatory effects of the complexes on starch digestion rate *in vivo* and the glycemic response mechanism.

## CRediT authorship contribution statement

**Yerkengul Yertai:** Writing – review & editing, Writing – original draft, Methodology, Investigation, Formal analysis, Data curation. **Zixiang Shao:** Investigation, Writing – review & editing. **Yutian Gao:** Investigation, Writing – review & editing. **Yidong Wei:** Investigation, Writing – review & editing. **Wenting Li:** Investigation, Writing – review & editing. **Liqiong Xie:** Writing – review & editing. **Torkun Mamet:** Writing – review & editing, Supervision, Methodology, Funding acquisition, Formal analysis, Data curation, Conceptualization.

## Declaration of competing interest

The authors declare that they have no known competing financial interests or personal relationships that could have appeared to influence the work reported in this paper.

## Data Availability

No data was used for the research described in the article.

## Appendix A Supplementary data

Supplementary material. Image 1
