# Supplementary material for: Starch-flavonoids interactions from Cyperus esculentus L. (tigernut) oil cake: insight on structural, physicochemical properties and digestibility
Source: Food Chem X. 2025 Sep 5;30:102999. doi: 10.1016/j.fochx.2025.102999 (PMC12491735; doi:10.1016/j.fochx.2025.102999)
Supplement: Supplementary file 1 — Supplementary material. [file mmc1.docx]

Dear Editor,

Subject: Request for Author Change for Manuscript "Starch-flavonoids interactions from Cyperus esculentus L(tigernut) oil cake：insight on structural, physicochemical properties and digestibility"

I hope this message finds you well. I am the corresponding author of the manuscript titled "Starch-flavonoids interactions from Cyperus esculentus L(tigernut) oil cake：insight on structural, physicochemical properties and digestibility" which was submitted to your esteemed journal on Aug 31, 2025(FOCHX-D-25-01881R1).

We would like to request the following changes to the author list:

Addition of Yutian Gao as the third author, as she has made significant contributions to this revision.

Author contributions are as follows:

Torkun Mamet(Corresponding author): Writing–review & editing, Writing–original draft, Supervision, Methodology, Funding acquisition, Formal analysis, Data curation, Conceptualization.

Yerkengul. Yertai(First author): Writing–review & editing, Writing–original draft, Methodology, Investigation, Formal analysis, Data curation.

Zixiang Shao(Second author): Writing– review & editing, Investigation, Formal analysis.

Yutian Gao(Third author): Writing–review & editing, Investigation, Data curation.

Yidong Wei(Fourth author): Writing–review & editing, Investigation.

Wenting Li(Fifth author): Writing–review & editing, Investigation.

Liqiong Xie(Sixth author): Writing–review &editing.

All authors, including the newly added author, have agreed to this change and understand the academic and ethical responsibilities associated with this decision. We have attached the consent signatures of all authors.

If you have any questions or specific procedures, please contact us.

Thank you for your consideration and support.

Sincerely,

All author signatures are as follows：
